# The Influence of New Bioactive Materials on Pulp–Dentin Complex Regeneration in the Assessment of Cone Bone Computed Tomography (CBCT) and Computed Micro-Tomography (Micro-CT) from a Present and Future Perspective—A Systematic Review

**DOI:** 10.3390/jcm11113091

**Published:** 2022-05-30

**Authors:** Mirona Paula Palczewska-Komsa, Bartosz Gapiński, Alicja Nowicka

**Affiliations:** 1 Department of Conservative Dentistry and Endodontics, Pomeranian Medical University in Szczecin, Powstanców Wielkopolskich 72, 70-111 Szczecin, Poland; nowicka6@gmail.com; 2 Division of Metrology and Measurement Systems, Institute of Mechanical Technology, Poznan University of Technology, Jana Pawła II 24, 60-965 Poznań, Poland; bartosz.gapinski@put.poznan.pl

**Keywords:** CBCT, direct pulp capping, micro-CT, tertiary dentin, total pulp amputation, bioactive material, pulp–dentin complex

## Abstract

The present paper is the first article providing a systematic literature review on the visualization of tertiary dentin influenced by modern bioactive materials in CBCT and micro-CT. Six database searches of studies on tertiary dentin visualization using CBCT produced 622 records in total, and the search of the studies on tertiary dentin using micro-CT produced 502 records in total. The results were thoroughly selected considering the inclusion criteria, and five research papers using CBCT and nine research papers using micro-CT for visualization of tertiary dentin were eventually qualified for the analysis. All the non-randomized and randomized studies presented good and high levels of quality evidence, respectively. Among the bioactive materials used, the most frequently analysed were: MTA, Biodentine dentin matrix hydrogel, Pro Root MTA, and EndoSequence root repair material. The highest thickness of the tertiary dentin was achieved after the use of MTA material in both imaging techniques. The remaining parameters had different results, taking into account the CBCT and micro-CT analysis. The possibilities of the qualitative and quantitative assessment of the particular parameters of tertiary dentin using CBCT and micro-CT techniques were presented and analysed. CBCT and micro-CT analyses can be useful in the assessment of tertiary dentin formed beneath the bioactive material applied during vital pulp treatment. The research argues that the presented results differ depending on the material applied to the pulp, the study duration (4–6 weeks), difference in teeth, species (rats, human), as well as the applied technique and differences in computer software used for the analysis.

## 1. Introduction

For many years, numerous preparations have been used as part of vital pulp therapy (VPT) with the aim of promoting the defence and regeneration capabilities of the pulp, thus leading to the healing of lesions. Therapeutic success is conditional upon the clinical situation and applied bioactive material [1]. Pathological conditions of the pulp–dentin complex show a dynamic spectrum subject to various biological and spatiotemporal factors. Consequently, it is a prerequisite that in the discussion on the regenerative processes both tissues are considered jointly instead of discussing the processes separately for dentin and pulp [2].

In the pulp chamber, odontoblasts (the cells forming the dentin) form a unicellular layer arranged in several rows. The dentin itself is an example of a rigid and durable natural biomaterial of exceptional durability. It consists of both soft and rigid components (proteins and mineral salts) at precisely arranged levels [3]. Odontoblasts form the predentin during the tooth development process and after its completion, termed the primary and secondary dentin, respectively [4]. Mantle dentin of a thickness of 100–200 μm forms around the predentin. It lacks dentinal tubules and shows lower mineral density [5]. Both the primary and secondary dentin encompass the peritubular, intertubular or intratubular dentin [6]. Secondary dentine (SD) first forms as teeth erupt, and the process lasts a lifetime. Attrition can also be a stimulus to induce secondary dentin deposition. It has been found that the effect of tooth wear on secondary dentin formation is not limited to the area subject to wear. Moreover, occlusal forces cause the deposition of secondary dentin below the enamel–dentin junction. This is a compensation mechanism to strengthen the tooth structure against deflection due to mechanical stress. The SD formation mechanism may be affected by occlusal loading, causing the activation of odontoblastic cells to form a more robust SD layer. Additionally, the age of the individual, rather than the attrition level, may affect SD accumulation in pulp chamber. [7]. In response to external stimuli, particularly due to caries, the pulp induces the formation of tertiary dentin. Mild stimuli lead to deposition of reactionary dentin synthesized by the present odontoblasts [4]. Reactionary dentin, apart from the teeth with a carious cavity, can also be observed in attrited, damaged teeth. Reactionary dentin is a defence mechanism against physiological factors, forming sclerotic tissue that reduces dentin permeability. It is a mechanism that protects the pulp against harmful irritation [7,8]. Nowadays, in asymptomatic teeth or those with reversible pulpitis, the tissues affected by caries (deep caries) are selectively removed to avoid injury or pulp exposure [2]. Remineralization of damaged dentin can be achieved through mineral salts provided by the pulp fluid and pulp capping with a bioactive material [4,9]. The term “bioactive endodontic material” is used because the new materials have a variety of chemical compositions; however, they all have one common capability, which is bioactivity. This implies releasing calcium ions, electroconductivity, production of calcium hydroxide, formation of an interfacial layer between the cement and dentinal wall and formation of apatite crystals over the surface of the material in a synthetic tissue fluid environment such as phosphate buffer saline [10]. The situation is different in the teeth affected by attrition, damaged teeth, and in the elderly. The irritation dentin caused by attrition was irregular in form and structure and did not seem to protect the pulp entirely from exogenous irritants. The odontoblast layer subjacent to the exposed dentinal surface was reduced and sometimes missing, and odontoblast nuclei were present in tubules of the irritation dentin. Islands of chronic inflammation, circulatory disturbances, and haemorrhages were seen in the affected regions. The pulps of older teeth showed a decrease in the cell population and an increase in the amount of collagen. For this reason, teeth with dentin exposed by attrition should not be used for evaluation of operative procedures and materials [8]. 

The regenerative and reparative processes are ongoing; however, in the case of a major damage exceeding the compensation capacity of the tissue (loss of dentin or pulp), the odontoblasts die [1]. It is believed that under favourable conditions, pulp stem cells or other cells differentiate into cells similar to odontoblasts to form reparative dentin. Unlike the reactionary dentin, reparative dentin shows an irregular, at times tubular, structure with occasional cell insertion [11]. The formation of reparative dentin is expected when the bioactive material is placed directly on the exposed or amputated pulp (direct pulp capping, pulpotomy) [1,9,12,13]. However, the character of the new hard tissue beneath the materials used for pulp capping is not fully recognized. Multiple perforations as well as imperfections are often found in the newly formed dentin bridges, which brings questions to the adequacy of the term “dentin bridge”. Recently, terms such as dentin-like, bone-like and reparative dentin bridge have been used to describe the newly formed hard tissue [14]. From the regenerative–biological perspective, this process is to be treated as pulp wound repair aimed at healing the pulp–dentin complex, however, without restoring the original tissue microanatomy [7,15]. In such a case, there is a formation of a new tissue (tertiary dentin) which, despite the fact that it is not an exact copy of the original, is physiologically sufficiently functional [4]. This is termed atypical regeneration and describes the formation of an incomplete tissue which can show some particular differences in comparison with the original tissue, e.g., the shape, size or structure [16]. Both pure regeneration as well as repair constitute the finalisation of the healing process; however, the repair results in a formation of a replacement tissue (cicatrisation) which, to varying degrees, can contain regenerative components [17]. However, when the microbiological load is excessive and the pulpitis is uncontrolled and destructive in nature, the tissue is at risk of necrosis and is unable to form tertiary dentin [7]. In such a case, vital pulp therapy involves the partial or complete amputation of the pulp [18,19,20].

The regenerative processes of the pulp–dentin complex were demonstrated in the literature on the subject with the use of various radiological techniques: point images in different projections and cone beam computed tomography (CBCT). CBCT imaging is the advanced imaging technique employing multidetector computed tomography. The maxillofacial CBCT was introduced in 1996 and allowed the first clinically practical technology for the representative application of 3D imaging for endodontic purposes [21]. CBCT imaging is conducted by means of a rotating platform or gantry equipped with an X-ray source and detector [22]. A more precise imaging technique can be achieved with the use of micro-computed tomography (micro-CT). This system offers a much better spatial resolution (even less than micrometre) than that of the previous imaging techniques relying on X-rays [23]. In this imaging method, the three-dimensional (3D) volume of the imaged tissue can be virtually divided into sections and correlated with the internal structures [24]. Using this technique, mineralized tissues, such as teeth and bones, can be successfully visualised, and the new generation micro-CT apparatus allows for in vivo imaging of small live animals [25]. Thus far, CBCT and micro-CT techniques have been applied, among others, to investigate enamel thickness, conduct tooth measurement, analyse root canal morphology and evaluate root canal preparation, craniofacial skeletal development and structure, mineral concentrations of teeth, osseointegration and bone–implant integration [25,26,27,28,29,30,31,32]. The possibilities offered by CBCT and micro-CT imaging allow for a clear visualisation of tertiary dentin and, consequently, the assessment of the success of vital pulp treatment using various materials.

The aim of the present paper is the systematic review of literature on tertiary dentin influenced by various bioactive materials imaging using CBCT and micro-CT, and the critical assessment of the possibilities as well as limitations of these techniques. The present paper is the first article providing a systematic literature review on the visualization of tertiary dentin after vital pulp therapy in CBCT and micro-CT. 

## 2. Materials and Methods

### 2.1. Protocol and Registration

This systematic review is reported according to the Preferred Reporting Items for Systematic Reviews and Meta-Analyses (PRISMA) [33]. It was registered in the International Prospective Register of Ongoing Systematic Reviews (PROSPERO) under the registration number CRD42022299190 entitled “Assessment of tertiary dentine in cone bone computer tomography (CBCT) and computer microtomography (micro-CT)—A Systematic Review”. The clinical question was formulated and organized using the Population, Intervention, Comparison, Outcome and Study (PICOS) strategy.

### 2.2. Review Questions

**Figure 1:**
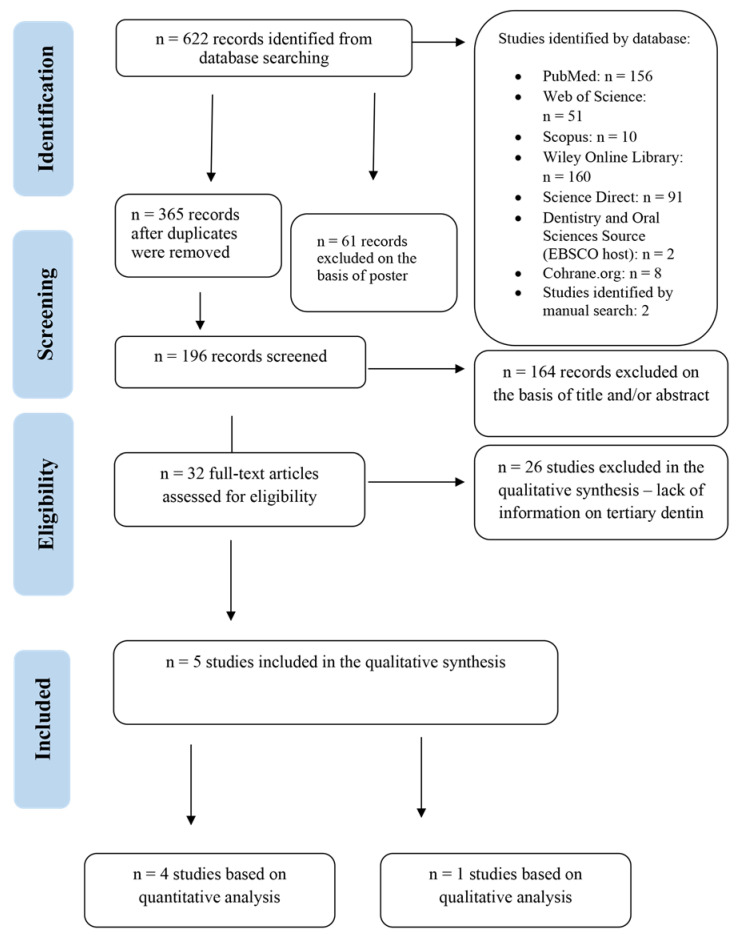
Preferred Reporting Items for Systematic Reviews (PRISMA) flow diagram of the search CBCT.

**Figure 2:**
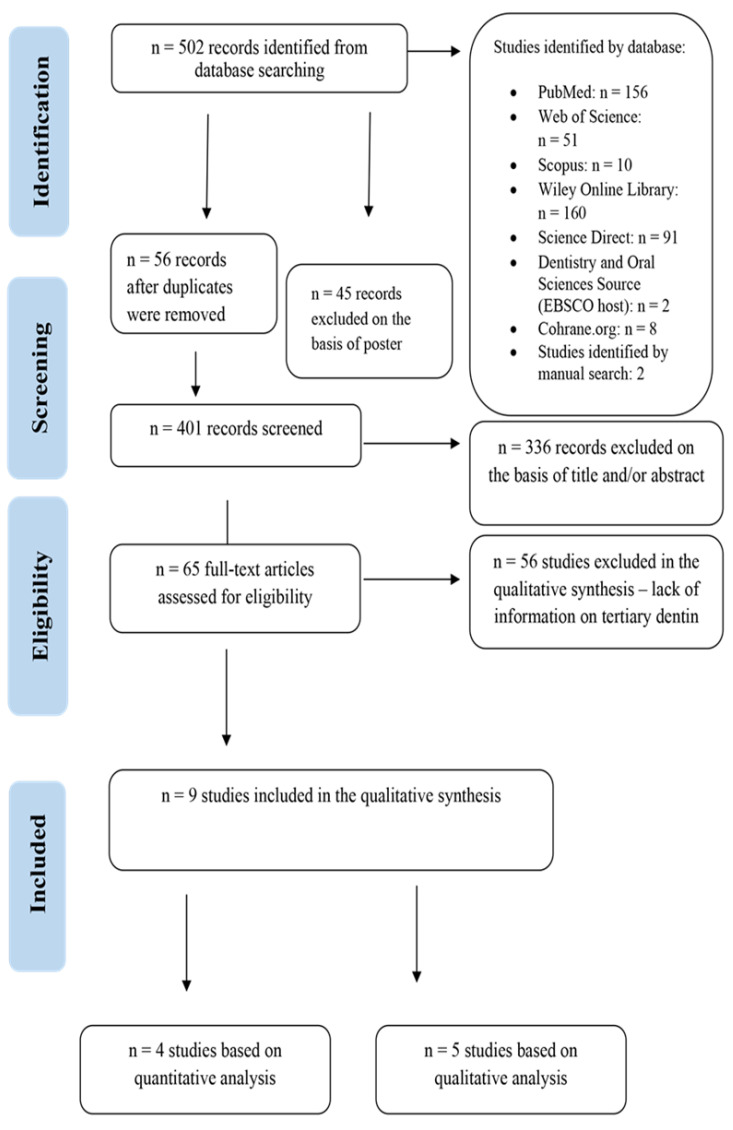
Preferred Reporting Items for Systematic Reviews (PRISMA) flow diagram of the search micro-CT.

The review of the literature was based on the Preferred Reporting Items for Systematic Reviews (PRISMA) (Figure 1 and Figure 2) [33].

The present article aims to answer the following questions:

- To what extent is tertiary dentin visualised using CBCT and micro-CT?
- What parameters of tertiary dentin can be measured using CBCT and micro-CT?
- What bioactive materials are most often used for vital pulp therapy?

### 2.3. Information Sources and Search Strategy

Six databases (PubMed/MEDLINE, Web of Science, Scopus, Dentistry and Oral Sciences Source (EBSCOhost, Wiley Online Library, Science Direct and Cohrane.org) were electronically searched by two independent reviewers (M.P-K. and B.G.) for publications containing information on CBCT and dentin or micro-CT and dentin. Any disagreements were resolved by mutual discussion and by reaching consensus with an experienced researcher (A.N.). Publications were searched with a year limit (2012–2021). The last search was conducted on 7 July 2021. The search phrases are presented in Table 1 and Table 2. All papers were imported into the Mendeley (2020 Mendeley Ltd., Elsevier, Rotterdam, The Netherlands) program for the management of scientific bibliography. Having removed the duplicates, all articles (titles, abstracts) were examined. Articles were extracted based on the inclusion criteria listed below:

- In vitro and in vivo studies that evaluated tertiary dentin in CBCT or micro-CT imaging.
- Publications concerning the properties of the dentin bridge in CBCT or micro-CT imaging.

### 2.4. Eligibility Criteria

The articles of which the full texts were not available in English were excluded. All exclusion and inclusion criteria are described in Table 3. Data concerning the papers, material, methods, the number of cases/controls, the type of clinician problem, observation time, the type of assessment and parameters of the dentin bridge were extracted from the selected papers by means of a standardized sheet in Microsoft Office Word 2010 (Microsoft Corporation, Redmond, WA, USA).

### 2.5. Risk of Bias in the Studies Included

The quality of in vivo studies was assessed with a modified methodological index for non-randomized studies (MINORS) modified by the authors [34]. In the MINORS scale (for in vivo studies), the following were taken into account: clear aim, clear protocol, inclusion of additional patients or animals, prospective data collection, justification of the sample size, observation period, endpoints relevant to the study purpose, blind analysis. The items were rated: 0, not reported; 1, introduced but inadequate; and 2, introduced and appropriate. Test quality classifications were rated as: poor (0–5), fair (6–10) and good (11–16) for in vivo tests [35]. 

The randomized studies were assessed using the data extraction and data assessment protocols modified after Guyot et al. (1993, 1994) with minor adjustments [36,37]. The data were extracted independently by the two reviewers (M.P-K and B.G.). The extracted data could be stratified into subsets according to study design, description of the subjects and materials used and outcome measures. Disagreements were resolved by discussion. Table 4 describes the risk of bias according to the MINORS scale in vivo studies (modified methodological index for nonrandomized studies), while Table 5 describes the risk of bias according to the levels of evidence and criteria for evidence synthesis for randomized studies.

## 3. Results

The studies eligible for the analysis were divided into two groups: non-randomized studies (*n* = 11) [14,38,39,40,41,42,43,44,45,46,47] and randomized studies (*n* = 3) [48,49,50]. All research articles under analysis were in vivo studies. 

### 3.1. Assessment of Studies

All non-randomized studies (*n* = 11) presented a good level of study quality and were included in further analysis. In addition, all randomized trials (*n* = 3) presented high level of evidence quality research and were included for further analysis. All studies were undertaken in healthy teeth; that is, there were no studies of teeth with carious exposures. One of fourteen studies had several follow-up periods. The follow-up periods of the included studies, regardless of level of evidence, varied between 30 days and 730 days. There were no reports of hard tissue barriers for observation periods under 4 weeks.

### 3.2. Results concerning Studies on Tertiary Dentin Visualisation Using CBCT

The database search produced 622 records in total. However, not all databases allowed the application of all the inclusion and exclusion criteria. Consequently, at a subsequent phase of selection, the research papers which did not meet the criteria were excluded by hand and the duplicates were eliminated. The results were thoroughly selected considering the inclusion criteria, and 5 research papers were eventually qualified to the analysis [38,39,40,48,50]. All studies (*n* = 5) were conducted on human teeth and were approved by the relevant Bioethics Committees. Two studies were conducted on premolars [38,40], whereas the remaining studies were conducted on molars [39,48,50]. In all of the studies under analysis, the teeth were free of caries, inflammation or periodontal disease. Additionally, one study concerned indirect pulp capping [50], and the methodology of four studies concerned direct pulp capping [38,39,40,48]. In CBCT studies, qualitative (visualization) and quantitative (radiodensity, thickness and volume) analyses of tertiary dentin were performed. Tertiary visualization was described in one study [40]. The most complete dentin bridges were observed after direct pulp capping (DPC) with mineral trioxide aggregate cement MTA and EndoSequence root repair material (EERM). Radiodensity of tertiary dentin was analysed on the basis of two articles [39,50]. It turned out that tertiary dentine after indirect pulp capping (IPC) with dentin matrix hydrogel (TDMH) (98%) showed the highest radiodensity, and the lowest was found after using it in IPC CH (78%). As for the thickness of the dentin bridge, it was analysed by the authors of three studies [39,48,50]. The thickest tertiary dentin was obtained after DPC with MTA material (0.540–0.930 mm), while the thinnest tertiary dentin layer was recorded after DPC for Single Bond Universal (SBU) (0.030 mm). The volume of tertiary dentin is given by the authors of two publications [38,48]. The highest volume of tertiary dentin was after DPC with Biodentine (BD) (1.1 mm^3^), and the smallest after DPC with SBU (0.07 mm^3^).

**Figure 3:**
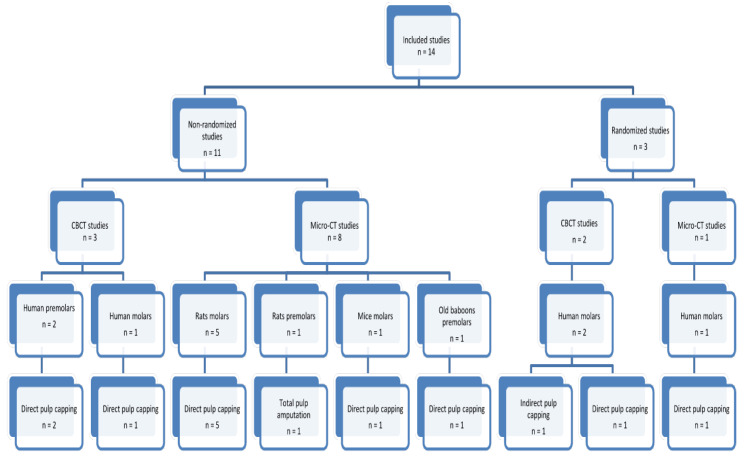
Diagram of the included studies in the analysis.

For ease of reference, Figure 3 and Table 6 summarize the research studies on the CBCT and dentin. Table 7 and Table 8 present the material under analysis, the most significant parameters regarding CBCT analysis as well as the methodology of the assessment and parameters (in unified units) of the newly formed hard tissue (tertiary dentin). 

### 3.3. Results concerning Studies on Tertiary Dentin Using Visualisation with Micro-CT

The database search produced 502 records in total. However, it must be emphasized that not all databases allowed for the application of all the inclusion and exclusion criteria. Therefore, at a subsequent phase of selection, the research papers which did not meet the set criteria were excluded by hand, and the duplicates were eliminated. The results were thoroughly selected with respect to the inclusion criteria, and nine research papers were eventually qualified for the analysis [14,41,42,43,44,45,46,47,49]. Among the studies under analysis, six were conducted on rat teeth [14,41,42,43,44,45], one on mice teeth [46], one paper concerned the teeth of old baboons [47], and one study was conducted on human teeth [49]. All of the studies (*n* = 9) were approved by the relevant Bioethics Committees. Two studies were conducted on premolars [45,47], whereas the remaining studies were conducted on molars (*n* = 7) [14,41,42,43,44,46,49]. In all of the studies under analysis, the teeth were free of caries, inflammation or periodontal disease. Additionally, eight studies concerned direct pulp capping [14,15,16,17,18,19,20,21,22,23,24,25,26,27,28,29,30,31,32,33,34,35,36,37,38,39,40,41,42,43,44,45,46,47,48,49], and the methodology of one study concerned total pulp amputation [45]. In micro-CT examinations, qualitative (visualization) and quantitative (thickness and volume) analysis of tertiary dentin was performed. Tertiary visualization is described in five studies [14,41,44,45,46]. The most mineralized tertiary dentin was observed after direct pulp capping (DPC) with Pro Root MTA with osteostatin (OST). As for the thickness of the dentin bridge, it was examined by the authors of two studies [47,49]. The thickest tertiary dentin was obtained after DPC with MTA material (0.78 mm), while the thinnest layer of tertiary dentin was obtained after DPC for calcium hydroxide (CH) material (0.22 mm). The volume of tertiary dentin is given by the authors of two publications [38,48]. The highest volume of tertiary dentin was after DPC with MTA with the addition of pre-reacted glass-ionomer (S-PRG) (0.05 mm^3^), and the smallest after DPC with EndoSequence Root Repair Material (iRoot BP Plus) (0.02 mm^3^).

For ease of reference, Table 6 summarizes the research studies on the micro-CT and dentin. Figure 3 and Table 7 and Table 8 present the material under analysis, the most significant parameters regarding micro-CT analysis as well as the methodology of the assessment and parameters (in unified units) of the newly formed hard tissue (tertiary dentin).

Due to high methodological heterogeneity of the studies under analysis, a meta-analysis was not conducted.

## 4. Discussion

The discussion is divided into smaller subsections to present the possibilities of the qualitative and quantitative assessment of the particular parameters of tertiary dentin using CBCT and micro-CT techniques. When referring to particular studies, the present analysis follows the original terms used therein, therefore instead of the term “tertiary dentin”, the visualised tissue is at times referred to as “dentin bridge”. Moreover, further on in the discussion section, limitations as well as prospects for future use of the analysed imaging techniques in vital pulp treatment are presented.

### 4.1. Visualization of Tertiary Dentin

The qualitative assessment, such as the visual determination of the parameters of tertiary dentin, is often used both in CBCT [40,41,46,48], as well as in micro-CT technique [14,44,45,49]. The visual analysis of the dentin bridge using CBCT relies on identifying the presence of lack of the visible dentin bridge, the presence of islands of calcified material as well as detecting the complete (continuous) dentin bridge [40,48]. Following in direct pulp capping of human teeth with calcium hydroxide (CH), mineral trioxide aggregate cement (MTA), Biodentine (BD) and Single Bond Universal (SBU), CBCT imaging conducted after 6 weeks showed 25 bridges out of the 37 histologically identified. The highest number of complete dentin bridges was recorded following the use of MTA and BD [48]. In another similar study on humans, 8 weeks after the application of CH, MTA, BD and EndoSequence Root Repair Material (ERRM), the highest number of complete dentin bridges was recorded in the premolar group following pulp capping with MTA and ERRM [40]. It was found that two thirds (67%) of MTA- and ERRM-treated teeth developed structures which show similarity to tubular dentin in the interface between the applied capping material and the pulp [40]. 

The micro-CT analysis was also conducted on the amputated pulp of 29 rats: the teeth were covered with lithium chloride (LiCl), and the formation of uniform dentin bridge in radiopacity was found [45]. On the reconstructed sagittal micro-CT images, no dentin bridges were identified in the control group. In contrast, there were complete dentin bridges over the surface of the exposed pulp in 11 out of 19 samples in the LiCl group. The inner dentin wall was used to define the pulp area. The great potential of LiCl as a bioactive mediating organ-specific trans differentiation is recognized. The formation of dentin bridges immediately under the treated regions was efficiently induced by the capping procedure with the use of LiCl. It transpires from the obtained results that LiCl may be an efficient tool for achieving regeneration of dentin in a novel treatment approach. Furthermore, it was suggested that the pulp capping procedure with the use of LiCl is advantageous as compared with the control in which pulp stumps were treated only with a mixture of macrogol and propylene glycol [45]. The reason for this is that, due to LiCl, a well-developed tubular formation was identified in the radiological findings. In the instances of covering the pulp stumps with other materials, the radiological image showed a tissue of worse osteodentin structural parameters. In another study, the pulp of molar teeth of 32 rats was treated with white mineral trioxide aggregate cement (ProRoot MTA) in combination with different osteostatin concentrations (OST) [14]. The formation of a reparative mineralized bridge in the residual pulp was found in all groups in micro-CT analysis. In comparison with the control in which the pulp was covered with ProRoot MTA without OST, it was found that the study groups showed more mineralized bridge [14]. The results imply the possibility of using OST as a supplementary capping material combined with MTA in order to achieve a synergistic effect in the formation of hard tissue. However, the said study does not provide the parameters, based on the radiological findings, of the newly formed dentin bridges. In yet another study on mice teeth, the pulp was capped with MTA. The newly formed hard tissue was identified in the form of radiopaque regions covering the exposed pulp surface. The area with high X-ray absorption in the m-CT images under the pulp-capping area on the 4th and 7th days is considered to be dentin fragments. On the 14th day, the reactionary dentin aligned with odontoblasts was formed under the pre-existing dentin. On the 28th day, the amount of reactionary dentin was increased. Additionally, the reparative dentin was formed in the pulp-capping area. Then, the dentin bridge covering the exposed pulp surface was considered to be complete [46]. However, for the purpose of a more precise determination of the parameters of tertiary dentin, apart from the radiological techniques which prove to be insufficient also concerning CBCT and micro-CT (owing to a small scale), numerous authors apply other techniques allowing for tissue assessment on a cellular level, such as: histologic analyses, in situ hybridization, cell culture and real-time RT-PCR analysis, immunohistochemical staining, double fluorescent staining or histomorphometric analysis. 

Generally, CBCT scanning is justified when the exact position of the tooth or its relationship with intimate anatomical structures and possible resorption of neighbouring teeth cannot be reliably assessed using 2D radiographs or if CBCT imaging will otherwise influence treatment planning [50,51]. In general, when referring patients for radiological examinations, including CBCT, it is imperative to follow the ALARA (as low as reasonably achievable) principle to minimize the effects of harmful X-ray radiation. A shift from ALARA and ALADA (as low as diagnostically acceptable) towards ALADAIP (as low as diagnostically acceptable, indication oriented and patient specific) is currently proposed [50]. One of the significant disadvantages of CBCT imaging is a longer scan time as compared with the conventional imaging. Additionally, the movement of a patient during scanning may result in image artefacts and may degrade the image quality [52]. This technique is not routinely recommended for the evaluation of the success of vital pulp treatment. However, conversely, it is indispensable in the diagnosis of pulp pathology, resorption, root fractures, complex anatomy of the root system and the identification of pathological changes in periapical tissues [52]. Furthermore, in endodontic imaging (also for the purpose of assessing the form of the tertiary dentin), higher spatial resolution may be required [52]. Although, theoretically, the spatial resolution of CBCT apparatus may be high due to a small size of focal spot, voxel, beam projection geometry, scattering, patient movement, detector motion unsharpness and fill factor, the number of projections and reconstruction algorithms, it also affects the final resolution and, consequently, the possibility of visualizing particular structures [53]. Since CBCT scanning may take from several to 45 s, the resulting image is susceptible to patient movement (even the heartbeat can result in head movement of 80–90 µm in length) [54]. Apart from clinical studies, CBCT technique is also applied in scientific studies on human teeth following the extraction. The disadvantage of CBCT imaging lies in the limitation due to the size of the analysed structures and, consequently, lack of the possibility of identifying details in small objects.

Much better parameters of structures and less image distortion is provided by micro-CT imaging. With the use of this technique, it is possible to identify the reference points in a precise manner and calculate parameters such as thickness or the volume of the visualised structure. However, it is still impossible to visualize the analysed structure on a cellular level. Furthermore, teeth visualisation using micro-CT technique takes as long as several dozens of minutes [43]. Owing to such a long exposure time and, consequently, the amount of radiation dose, this technique is not to be used in vivo in patients (a tooth that functions in the patient’s mouth), but indirectly in vivo, the extracted teeth can be visualized with it [53].

The selection of teeth for the analysis of tertiary dentin formation after the use of bioactive pulp-covering materials is extremely important. Apart from the fact that these teeth should be free from caries, they should not be exposed to occlusive stress, should not be abraded, and should not be taken from old people. These factors irritate the pulp and induce its return mechanisms in the form of reactive dentin deposition [7,8]. This dentin is also a form of tertiary dentine, however; in contact with bioactive material, it has a chance to form only in the indirect pulp-capping procedure [4].

Additionally, it must be observed that in order to conduct an accurate assessment of the characteristics and functionality of the tertiary dentin, both the qualitative and quantitative analyses of the visualized structure are to be taken into consideration. Such an analysis should also be correlated with the histological analysis and, in the case of in studies on human, with the analysis of the clinical condition. Only such a combination of studies may provide a full answer to the question of whether the newly formed dentin adequately fulfils its function.

### 4.2. Areas of Newly Formed Tertiary Dentin

In the study conducted on 10 molar teeth of rats, after 4 weeks of observation, the areas of the newly formed reparative dentin as well as the pulp cavity were measured in five transverse sections selected at random. In addition, the relative ratio of reparative dentin to pulp cavity was calculated [44]. Following reconstruction, colour images displaced grey images in order to visualise mineral density. In a normal dental tissue, dentin and bone are represented by a green colour, enamel is represented by a blue colour and pulp and soft tissue by a purple colour. The superior formation of tertiary dentin in a visual assessment was found in teeth in which direct pulp capping was performed with MTA. The explicit mechanisms of formation of the dentin bridge by MTA is not fully known. Pulp capping with the use of MTA affected the pulp cells cytologically and functionally, thus resulting in the formation of fibrodentin and reparative dentin. A slightly inferior formation of the tissue was observed following pulp capping with Biodentine and BioAggregate (BA). Moreover, it transpires that in terms of characteristics, the newly formed reparative dentin due to Biodentine and BioAggregate resembled osteodentin more closely. It is indicated by the obtained results that new reparative dentin was induced by MTA, BD and BA, and that the characteristics of MTA-induced dentin were superior [44]. 

The visualisation and qualitative assessment of tertiary dentin surface is subjective, inadequate in its entirety and, generally, provides little information on the quality of tertiary dentin. Consequently, it is to be considered as an auxiliary assessment which can provide information on the quality of the formed tertiary dentin only when combined with other parameters.

### 4.3. Thickness of Tertiary Dentin

There are numerous studies on tertiary dentin thickness following indirect and direct pulp capping with different materials using CBCT technique [39,48,50] and micro-CT technique [47,49]. In the CBCT visualisation of 95 teeth of children, the difference between the average distances from beads to the roof of the pulp chamber was taken to be the thickness of reparative dentin [55]. The clinical study on humans showed, as demonstrated with CBCT, that following the indirect pulp capping with MTA, glass ionomer cement (GIC) type VII and CH, the thickness of tertiary dentin (reactive dentin in this case) was the highest following the use of MTA [55]. In addition, in the direct pulp capping with dentin matrix hydrogel (TDMH), BD or MTA, the highest tertiary dentin thickness was observed for MTA [39]. Similarly, CBCT visualisations following the extraction of teeth treated with direct pulp capping using CH_,_ MTA, BD and SBU, showed that the highest mean thickness of tertiary dentin was found in the MTA group, whereas the highest maximal thickness was in the BD group [48]. The research argues that MTA and BD have a markedly higher stimulatory activity on pulp cells, as compared with CH, which results in thicker reparative dentin bridges.

The studies employing micro-CT technique on old baboons teeth, the exposed pulp of which was covered with CH, Pro Root white MTA or white Portland cement (PC) demonstrated that the thickest layer of tertiary dentin was obtained following pulp capping with Pro Root White MTA [47]. Hard tissue apposition was found to be minimal and incomplete in the CH group. At high magnification, the reparative hard tissues were identified as a tubular, showing uneven thickness, with porosities and tunnel defects. The thickness of the reparative hard tissue was 0.22–0.024 mm. The reparative hard tissues formed following the application of ProRoot white MTA for 4 months appeared to be thicker and less porous, and the thickness was found to be 0.43–0.053 mm [47]. Nonetheless, there were tunnel defects within the tubular hard tissues identified with higher magnification images. The thickness of reparative hard tissues which formed following PC application for 4 months was similar (0.4–0.058 mm) to the thickness obtained with the application of ProRoot white MTA. Similar to other groups, the reparative hard tissues were found to be tubular and showed no resemblance to physiologic secondary dentin or reactionary tertiary dentin. At high magnifications, it was possible to identify porosities as well as tunnel defects containing granulation tissues. The formation of moderately radiopaque reparative hard tissue under the radiolucent PC was confirmed with micro-CT [47]. In yet another study [49], the exposed pulp of human premolars was treated with betamethasone/gentamicin (BG) cream and MTA. Eight weeks later, following pulp capping, the teeth were extracted and analysed with micro-CT. The thickest tertiary dentin tissue was found in teeth covered with MTA. Furthermore, 89% of the specimens showed the formation of a dentin bridge at the exposure site. The average thickness of reparative hard tissue was determined at 78.50 um; 67% of the specimens showed a formation of a complete dentin bridge with a normal structure; 23% demonstrated a partial or incomplete hard tissue barrier formed at the exposure site. At the exposure site, hard tissue formed in the form of diffuse calcification in four samples treated with MTA. The performance of MTA was found to be superior to that of BG cream. It is argued that the inferior performance of BG cream may be due to the consistency as well as to different chemical composition. Due to its form, i.e., a cream, BG dissolves in tissue fluids and does not provide a solid surface. Conversely, even in the presence of moisture, MTA sets to a hard structure and provides a solid base and sealing ability. The drawbacks such as high solubility and fast dissolution also apply to conventional calcium hydroxide in the process of reparative dentinogenesis [49]. 

Overall, when conducting studies on determining the thickness of tertiary dentin, the following criteria are to be taken into consideration: study period which in the studies under analysis ranged from 2 weeks to 2 years, the type of pulp capping and consequently the type of the formed dentin (reactive or reparative), as well as the species (human, old baboons). The only common denominator of the analysed papers is the fact that the studies were conducted on molars and the use of MTA. In the case of MTA application, the thickness of tertiary dentin, depending on a given study, ranged from 7.19 um (after 2 weeks) to 0.93 mm (after 2 years).

### 4.4. Tertiary Dentin Volume

The volume of tertiary dentin (DV) was assessed both in CBCT studies [48] and in micro-CT studies [41,42,43]. The study employing CBCT of extracted human teeth (after 6 weeks of studies) showed that tomography resolution limited the minimum measurable distance, and that it was possible to measure the small bridges only on histologic images [48]. In the CH group, the volume of the formed reparative dentin was found to be moderate and in the MTA and BD group, moderate to high, without any marked differences identified between the two latter groups. Only two bridges of small volume and radiolucent tunnel defects were found in the SBU group. The result was conditioned by the cytotoxic nature of SBU following its application to the pulp. Notably, the reparative dentin bridges in the BD group showed the highest average and maximum volumes in comparison with the other three groups. BD and MTA resulted in the formation of bridges with a significantly higher average volume as compared with SBU. As has been observed [48], determination of the precise location as well as the measurement of the volume of dentin bridges using CBCT images is difficult when not correlated with histologic findings owing to certain limitations of CBCT imaging, such as a low contrast, background noise, and a small area of evaluated tissue.

In the study conducted on an animal model (rats) with the use of micro-CT, for the purpose of the assessment of tertiary dentin volume (DV), defined as the tertiary dentin area without any necrotic area, defect, or cellular content and, additionally, to determine the tissue volume (TV), defined as the volume of tertiary dentin including defects or cellular contents and compactness of tertiary dentin DV/TV ratio, standard mineral reference phantom was used (Ratoc System Engineering) [43]. It was demonstrated that the volume of bridges after 2 and 4 weeks of study was comparable for ProRoot MTA and iRootBP Plus, yet after 4 weeks, the iRoot BP Plus group showed a markedly higher DV/TV values, in comparison with the ProRoot MTA group. The three-dimensional radiographic assessment used in this study allowed for observation of the entire tertiary dentin structure in a panoptic view with a high resolution of 7.1 μm on individual images. The quality and quantity of tertiary dentin were also evaluated by calculating micro-CT parameters using a standardized phantom. Detection of small defects was possible due to high-resolution micro-CT images, and a decreased integrity of ProRoot MTA specimens (3/5 samples) was found. Conversely, there were no defects in tertiary dentin in iRoot BP Plus samples. One ProRoot MTA specimen showed a complete tunnel-shaped defect on micro-CT analysis [43]. 

Similarly, another micro-CT analysis of the total dentin volume (BV) and tissue volume (TV) ratio (BV/TV) of tertiary dentine of rat teeth following pulp capping with CH, MTA and BD demonstrated that hard tissue formation was present for all the materials [41]. After 4 weeks, the hard tissue formed beneath each of the applied capping material was evaluated. Micro-CT showed that the formation of hard tissue was found in the CH-, MTA- and BD-treated teeth, whereas the teeth from the control showed no true dentin bridge formation. The total BV/TV ratio at the level of the mechanical pulp exposure showed significant differences between the groups. In comparison with the control group, the MTA- and BD-treated group exhibited markedly higher BV/TV ratios However, no significant differences were identified regarding the BV/TV ratio between the CH treated and control groups [41]. 

In general, determining the volume of dentin bridge is difficult using CBCT and micro-CT techniques, as the dentin bridge is a highly heterogeneous structure. Isolation of the whole field of the dentin bridge is complicated and time-consuming. Consequently, this may be the reason that the said parameter is only sporadically calculated in scientific articles. 

### 4.5. Radiodensity of Tertiary Dentin

CBCT and micro-CT are highly sensitive techniques allowing for the characterization and quantitative determination of dentin mineral density.

The application of CBCT technology provides accurate structural details and allows for evaluation of the radiodensity of the tertiary dentin in terms of Hounsfield Unit (HU) [56]. Enamel, dentin, and cementum have specific values which are >1500 HU, 1000–1500 HU, and <1000 HU, respectively [57]. The clinical study on humans was conducted using CBCT analysis of radiodensity of tertiary dentin 6 months after indirect pulp capping (IPC) with the use of various materials (CH, GIC, VII and MTA) [55]. The highest radiodensity was obtained for tertiary dentin following capping with MTA (984 HU). In turn, the mean percentage gain of radiodensity tertiary dentin at site, in comparison to healthy dentin, was 84% for this material. There was a slightly lower percentage of radiodensity tertiary dentin at site, as compared to healthy dentin; 78% was obtained for MTA in direct pulp capping (DPC) after 2 years of observation—also with the use of CBCT [39]. The CBCT study by Nowicka et al., 2015, measured the mineral density of mature dentin and tertiary dentin in patients (19–32 years of age) depending on capping of the pulp with different materials: (CH, MTA, BD and SBU), which indicates that after 6 weeks of observation, the mineralization of the formed tertiary dentin was almost half of that of mature dentin [48]. 

Most likely, the results obtained in the aforementioned studies are connected with the type of pulp capping, observation time, histological structure and mineralization processes of dentin. Dentin is approximately 70% mineral by weight; however, mineral content changes depending on the location within dentin, age, maturation, and disease processes [58]. Newly secreted dentin (predentin, young dentin) is unmineralized and consists of collagen, glycoproteins, and proteoglycans. It is similar to osteoid in bone and shows the highest thickness during dentinogenesis. Over time, predentin undergoes mineralisation (mature dentin). In turn, reparative dentin is implicated in the formation of a bone-like or in structure-less mineralization (pulp diffuse mineralization or pulp stones) and resembles bone more closely than dentin [59]. This can explain why reparative dentin is the least mineralised dentin structure. It was found that the highest and lowest average densities of tertiary dentin (reparative dentin, dental bridge) were recorded in the MTA (1253.9 mg/cm^3^) and SBU groups (1076.0 mg/cm^3^), whereas the values in the CH and BD groups were found to be similar (1000 mg/cm^3^) [48]. The study indicates that the density of tertiary dentin in the groups was also determined by the content of particles of the analysed materials present in the tissue, particularly the particles of SBU. The respective values provided by the aforementioned studies are presented in Table 7 and Table 9.

In a study conducted on adult rats, mineral density of tertiary dentin following the application of various bioactive materials during direct pulp capping was determined using micro-CT technique [42,43]. The lowest mineral density of tertiary dentin was found in the group of rats with the pulp capped with ProRoot MTA (1100 mg/cm^3^), whereas the highest values of dentin mineralization was identified in the group of rats with the pulp capped with pre-reacted glass-ionomer (S-PRG) (1500 mg/cm^3^). S-PRG is a material which has a protective effect against enamel and dentine demineralization [60,61]. Conversely, the modified S-PRG containing lithium ions may promote the formation of reparative tertiary dentin [62]. 

Summarizing the studies on mineral density of tertiary dentin, it can be concluded that the presented results differ depending on the material applied to the pulp. The area with high X-ray absorption radiopacity and density of the discussed material particles which are also present in tertiary dentin is essential [63]. Moreover, the mineral density of tertiary dentin can be conditioned by factors such as: different study duration (4–6 weeks), different teeth (the third and first molars), species (rats, human), the applied technique (CBCT and micro-CT) as well as different selection of measurement points and differences in computer software used for the analysis of tissue density. Additionally, mineral density of tertiary dentin is not a decisive parameter in the assessment of the degree of dentin regeneration following vital pulp treatment. Although the mineral density of tertiary dentin was relatively lower following the application of BD or CH on the pulp, the reparative tissues induced by MTA and BD were homogeneous. The tissues induced by CH were found to be porous, which implies that a reparative process is different from that induced by calcium silicate cements [48]. 

All numerical values together with the most important conclusions regarding the quantitative assessment of tertiary dentin with the use of CBCT and micro-CT are presented in Table 8. The quantitative micro-CT assessment is a complex technique, and the differences regarding the selection of measurement points of the selected parameters of tertiary dentin pose additional problems with interpretation and comparing the data with the results obtained by other authors.

### 4.6. General Future Aspects of Using X-ray Techniques in Tertiary Dentin Imaging

Recently, a new 3D imaging technique has been further developed, i.e., nano computer tomography (nano-CT). Owing to the smaller size of the focus spot of the X-ray in nano-CT, as compared with CBCT and micro-CT, this technique can visualize details on a smaller length scale [64]. With the decreasing size of the voxel and increasing spatial resolution, the field of view, i.e., VOI size, generally decreases. Consequently, for the visualization of bioactive materials and tissue structure in nanoscale, where the voxel size may be <100 nm, in the advanced commercial stationary nano-CT systems, usually a markedly lower volume, less than 1 mm^3^, is required [65]. Possibly in the future, this technique will be used to visualize tertiary dentin in teeth following vital pulp treatment and will replace the time-consuming histological techniques. Nonetheless, similarly to micro-CT, nano-CT is not to be used in vivo in humans due to exposure time and the amount of radiation dose.

## 5. Conclusions

1. CBCT and micro-CT analyses can be useful in the assessment of tertiary dentin formed beneath the bioactive material applied during vital pulp treatment. The limitations of micro-CT studies that can only be carried out in vivo indirectly, i.e., on extracted teeth, should be taken into account.
2. In CBCT and micro-CT analyses, the formed tertiary dentin is sufficient for determining whether the biomaterial applied to the pulp is effective in producing new hard tissue. The quality and quantity of tertiary dentine depends on the type of: pulp capping, bioactive materials applied to the pulp, observation time, tooth type and species. The conducted research shows that bioactive calcium silicate cement contributes to the formation of tertiary dentin, which is superior compared to calcium hydroxide.
3. Most studies employ a qualitative analysis which does not require calibration phantoms and is less time-consuming by far, more often than the quantitative analysis. However, unlike the quantitative technique, this technique is subjective.
4. The fundamental limitation of micro-CT is the exposure time and the amount of radiation dose which prevents the visualisation of human teeth in the patient’s oral cavity and allows for it only following the extraction of teeth.

## Figures and Tables

**Table 1:** The CBCT search phrases.

Database	Filters		Search Phrases
Medline (PubMed) (156)	Free full text Full text Clinical Trial Journal Article Randomized Controlled Trial Meta-Analysis Review Systematic Review Last 10 years	1	(cone bone tomography) OR (CBCT)
		2	((Dentin) OR (Dental bridge)) OR (Dental pulp)
		3	Formation
		ALL	(cone bone computed tomography OR CBCT) AND (Dentin OR Dental bridge OR dental pulp) AND (Formation)
Web of Science (25)	Web of Science Categories: DENTISTRY ORAL SURGERY MEDICINE Document Types: ARTICLE Last 10 years	1	TOPIC: TS= cone bone computed tomography OR TS = CBCT
		2	(TS = (dentin) OR TS = (dental bridge) OR TS = (dental pulp))
		3	(TS = (formation))
		ALL	TS = (cone bone computed tomography) OR TS = (CBCT) AND ((TS = (dentin) OR TS = (dental bridge) OR TS = (dental pulp)) AND TS = (formation)
Science Direct (91) Scopus (49) Wiley Online Library (271) Dentistry and Oral Sciences Source (19)	Subject areas: Medicine and Dentistry Article type: Research articles Review Last 10 years Open access Language: English	1 2 3 ALL	cone bone computed tomography OR CBCT Dentin OR Dental bridge OR Dental pulp Formation (cone bone computed tomography OR CBCT) AND (Dentin OR Dental bridge OR dental pulp) AND (Formation)
Cochrane.org (8)	Subject areas: Dentistry and oral health Article type: Cochrane reviews Cochrane Central Register of Controlled Trials Jan 2012 and Jul 2021, in Cochrane Reviews, Trials	1 2 3 ALL	cone bone computed tomography OR CBCT “Dentin” “Dentin bridge” Formation (cone bone computed tomography OR CBCT) AND (Dentin OR Dental bridge) AND (Formation)

**Table 2:** The micro-CT search phrases.

Database	Filters	No.	Search Phrases
Medline (PubMed) (156)	Free full text Full text Clinical Trial Journal Article Randomized Controlled Trial Meta-Analysis Review Systematic Review Last 10 years	1	(micro computed tomography) OR (micro CT)
		2	((Dentin) OR (Dental bridge)) OR (Dental pulp)
		3	Formation
		ALL	(Micro computed tomography OR Micro CT) AND (Dentin OR Dental bridge) AND (Formation)
Web of Science (51)	Web of Science Categories: DENTISTRY ORAL SURGERY MEDICINE Document Types: ARTICLE Last 10 years	1 2 3 ALL	TOPIC: (TS = (micro computed tomography) OR TS = (micro CT)) (TS = (dentin) OR TS = (dental bridge) OR TS = (dental pulp)) (TS = (formation)) (TS = (micro computed tomography) OR TS = (micro CT)) AND (TS = (dentin) OR TS = (dental bridge) OR TS = (dental pulp)) AND (TS = (formation))
Science Direct (91) Scopus (10) Wiley Online Library (160) Dentistry and Oral Sciences Source (26)	Subject areas: Medicine and Dentistry Article type: Research articles Review Last 10 years Open access Language: English	1 2 3 ALL	micro computed tomography OR micro CT Dentin OR Dental bridge OR Dental pulp Formation (Micro computed tomography OR Micro CT) AND (Dentin OR Dental bridge) AND (Formation)
Cochrane.org (11)	Subject areas: Dentistry and oral health Article type: Cochrane reviews Cochrane Central Register of Controlled Trials Jan 2012 and Jul 2021, in Cochrane Reviews, Trials	1 2 3 ALL	micro computed tomography OR micro CT “Dentin” “Dentin bridge” Formation (micro computed tomography OR CBCT) AND (Dentin OR Dental bridge) AND (Formation)

**Table 3:** Exclusion and inclusion criteria.

Criteria	Included	Excluded
Full text	Available	Unavailable
Publication language	English	Other
Type of publication	Journal article	Books, documents
Type of research	Clinical trail Randomized controlled Research articles Review Meta-analysis Case Report	-
Subject area	Dentistry and oral surgery medicine	Other
Publication stage	Final, in press	Other

**Table 4:** Risk of bias according to the MINORS scale in vivo studies (modified methodological index for nonrandomized studies).

	Clear Aim	Clear Protocol	Inclusion of Consecutive Patients, Animals	Collection of Data	Justification of Sample Size	Follow-Up Period Appropriate to the Aim of the Study	Endpoints Appropriate to the Aim of the Study	Blinded Analysis	Study Quality
CBCT									
Bui et al., 2021 [38]	2	2	2	1	0	2	2	0	good
Holiel et al., 2021 [39]	2	1	2	2	0	2	2	0	good
Muruganandhan et al., 2021 [40]	2	1	2	2	0	2	2	2	good
Micro-CT									
Yoon et al., 2021 [14]	2	2	2	2	0	2	2	0	good
Yaemkleebbua et al., 2019 [41]	2	2	2	2	0	2	2	0	good
Okamoto et al., 2019 [42]	2	2	2	2	0	2	2	2	good
Okamoto et al., 2018 [43]	2	1	2	2	0	1	2	2	good
Kim et al., 2016 [44]	2	1	2	2	0	2	2	0	good
Ishimoto et al., 2015 [45]	2	2	2	2	0	2	2	0	good
Hara et al., 2021 [46]	2	1	2	2	0	2	2	0	good
Al-Hezaimi et al., 2011 [47]	2	2	2	2	0	2	2	2	good

Numbers coding: 2, introduced and adequate; 1, introduced but inadequate; 0, not reported.

**Table 5:** Risk of bias according to the levels of evidence and criteria for evidence synthesis for randomized studies.

High Level of Evidence:
The study was judged to have a high level of evidence if it fulfilled all of the criteria below: There was a sufficiently large sample of patients and teeth to detect a treatment effect, preferably calculated by a power analysis. The study was a true prospective experiment in which investigators randomly assigned the sample of patients and teeth to one or more intervention groups and a control group. The sample was described such that the pulp status was clear. The study personnel were blinded regarding the intervention. The procedure for performing the pulp capping was described in sufficient detail regarding the size, type and site of exposure as well as materials used, to permit replication. There was a proper account of the patients and teeth that entered the trial and attributed to its conclusion. The analysis of the hard tissue formation and status of the pulp were adequate; that is, the criteria were specified in the text or with a reference. The results were well documented and presented in terms of relevant data.
**Moderate Level of Evidence:**
A study was judged to have a moderate level of evidence if any of the above criteria was not met. Conversely, the study was judged not to have deficits that are described for studies with a low level of evidence.
**Low Level of Evidence:**
A study was judged to have a low level of evidence if it met any of the following criteria: There was not a sufficiently large sample of patients and teeth. There was not a randomization process. The sample and procedure were not described in sufficient detail to permit replication.

**Table 6:** Summary of the research studies on the CBCT, micro-CT and dentin.

Authors	Type of Research	Methods	Species	Pulp Exposure	The Material Used for the Pulp	Examined Teeth Sample Size (*n*)	Experiment Time
**Analysis CBCT**							
Mathur et al., 2016 (India) [50]	Randomized Controlled Trial	CBCT, Clinical examination	Human (children)	IPC	CH (setting type), GIC (Type VII), MTA	Primary second molars, Permanent first molar *n* = 95	2 y
Bui et al., 2021 (Vietnam) [38]	Research Study	CBCT Intraoral radiograph,	Human	DPC	BD	Premolars *n* = 11	9–12 w
Holiel et al., 2021 (Egypt) [39]	Comparative Study	CBCT Clinical examinations,	Human	DPC	TDMH, BD, MTA	Posterior teeth *n* = 45	2 y
Nowicka et al., 2015 (Poland) [48]	Randomized Controlled Trial	CBCT Clinical examination, CBCT Intraoral radiograph, Histological sections	Human	DPC	CH, MTA, BD SBU	Maxillary and mandibular third molars *n* = 44	6 w
Muruganandhan et al., 2021 (India) [40]	Comparative Study	CBCT, Histological sections	Human	DPC	CH, MTA, BD ERRM	Premolars *n* = 60	8 w
**Analysis micro-CT**							
Yoon et al., 2021 (Korea) [14]	Comparative Study	Micro-CT, Histological analysis, immunofluorescence staining	Sprague– Dawley Rats	DPC	ProRoot MTA, OST 100 μM + ProRoot MTA, OST 10 mM + ProRoot MTA	Maxillary molars *n* = 32	4 w
Yaemkleebbua et al., 2019 (Thailand) [41]	Comparative Study	Micro-CT, Histological analysis, immunofluorescence staining	Rats	DPC	CH, MTA, BD	Maxillary molars *n* = 32	4 w
Okamoto et al., 2019 (Japan) [42]	Research Study	Micro-CT, scanning electron microscopy (SEM), micro-X-ray fluorescence (μXRF)	Rats	DPC	S-PRG, MTA	First molars *n* = 32	4 w
Okamoto et al., 2018 (Japan) [43]	Comparative Study	Micro-CT, histologic analyses	Wistar Rats	DPC	ProRoot MTA, iRoot BP Plus	First molars *n* = 9	4 w
Kim et al., 2016 (Korea) [44]	Research Study	Micro-CT, histologic analyses immunohistochemical analysis using dentin sialoprotein (DSP)	Sprague- Dawley Rats	DPC	ProRoot MTA, BD, BA	First molars *n* = 10	4 w
Ishimoto et al., 2015 (Japan) [45]	Research Study	Micro-CT, histologic analyses, In situ hybridization, Cell culture and real-time RT-PCR analysis	Sprague- Dawley Rats	TPA	LiCl	First premolars *n* = 45	6 w
Hara et al., 2021 (Japan) [46]	Research Study	Micro-CT, Histological analysis, Immunohistochemical staining, Double fluorescent staining	Old male mice	DPC	MTA	First molars	28 d
Al-Hezaimi et al., 2011 (Saudi Arabia) [47]	Comparative Study	Micro-CT Histomorphometric analysis	Old baboons	DPC	CH, (PC)	Premolars *n* = 30	4 m
Alshwaimi et al., 2016 (Saudi Arabia) [49]	Randomized Controlled Trial	Micro-CT scanning and histologic analyses	Human	DPC	BG MTA	First molars *n* = 18	8 w

Abbreviations: BA, BioAggregate cement; BG, betamethasone/gentamicin cream; BD, Biodentine; CH, calcium hydroxide; DPC, direct pulp capping; DSP, dentin sialoprotein; EERM, EndoSequence root repair material; GIC, glass ionomer cement; IPC, indirect pulp capping; iRoot BP Plus, EndoSequence Root Repair Material; LiCl, lithium chloride; MTA, mineral trioxide aggregate cement; OST, osteostatin; PC, ProRoot white mineral trioxide aggregate, white Portland cement; ProRoot MTA, white mineral trioxide aggregate cement; S-PRG, pre-reacted glass-ionomer; SBU, Single Bond Universal; TDMH, dentin matrix hydrogel; TPA, total pulp amputation.

**Table 7:** The most important parameters of the CBCT and micro-CT apparatus.

	Analysis CBCT						
Source	Apparatus		Apparatus Parameters				
		Voltage (kV)	Current (µA)	Field of View (cm)	Voxel Size (mm^3^)	Slice Dimensions (Pixels)	Thickness of the Cut Layer (mm)
Bui et al., 2021 (Vietnam) [38]	ProMax^®^ 3DX-ray units Planmeca, Helsinki, Finland	90	140	5 × 5	0.10	1024 × 1024	
Holiel et al., 2021 (Egypt) [39]	CBCT imaging D-CBCT i-CAT FLEX, KaVo, Germany	90	90	5 × 5	0.12	-	20
Mathur et al., 2016 (India) [50]	CBCT i-CAT; Imaging Sciences International, Hatfield, PA, USA	-	-	13 × 16	0.5	-	12
Muruganandhan et al., 2021 (India) [40]	No data	-	-	-	-	-	-
Nowicka et al., 2015 (Poland) [48]	Cranex 3D, No. SE 1100155, Software Version Scanora 5.1.0.9; Soredex, Tuusula, Finland	Exposure parameters were standardized for each patient					
	**Analysis micro-CT**						
**Source**	**Apparatus**		**Apparatus parameters**				
		**Voltage (kV)**	**Current (mA)**	**Rotation** **(°)**	**Voxel size** **(mm^3^)**	**Filter** **(mm)**	**Exposure time** **(min)**
Yoon et al., 2021 (Korea) [14]	Micro-CT system SkyScan 1172, Brucker, Aartselaar, Belgium	70	141	180	-	0.5 Aluminium	-
Yaemkleebbua et al., 2019 (Thailand) [41]	Micro-CT 35, Scanco Medical, Brüttisellen, Switzerland	70	114	-	0.10	-	-
Okamoto et al., 2019 (Japan) [42]	Micro-CT scanner R-mCT2 Rigaku, Tokyo, Japan	90	160	-	0.10	-	3
Okamoto et al., 2018 (Japan) [43]	Micro-CT scanner SMX100CT Shimadzu, Kyoto, Japan	50	150	-	0.71	-	20
Kim et al., 2016 (Korea) [44]	Micro-CT system SkyScan 1172, Brucker, Aartselaar, Belgium	70	141	180	-	0.5 Aluminium	-
Ishimoto et al., 2015 (Japan) [45]	Micro-CT system InspeXio SMX-90CT Shimadzu, Kyoto, Japan	90	119	-	0.17	-	-
Hara et al., 2021 (Japan) [46]	No data	-	-	-	-	-	-
Al-Hezaimi et al., 2011 (Saudi Arabia) [47]	Micro-CT system SkyScan1172; Brucker, Kontich, Belgium	110	96	-	0.37	1 Aluminium	-
Alshwaimi et al., 2016 (Saudi Arabia) [49]	Micro-CT system SkyScan1172; Brucker, Kontich, Belgium	70	139	360	0.89	0.5 Aluminium	-

**Table 8:** Analysis of tertiary dentin in CBCT and micro-CT.

Tertiary Dentin					
Source	Quantitative Assessment	Qualitative Assessment			Conclusion
		Radiodensity (Mean Percentage Gain of Radiodensity at Site as Compared to Healthy Dentin %)	Thickness (mm)	Volume (mm^3^)	
**Analysis CBCT**					
Bui et al., 2021 [38]	-	-	-	BD Extracted teeth 1.1009 BD Real patient teeth 0.6186	BD could induce the formation of reparative dentin in direct pulp capping. The CBCT scan was the reliable modality for evaluation of dentin bridge formation.
Mathur et al., 2016 [50]	-	CH 78	CH 0.490	-	All three dental materials tested, i.e., CH(setting), GIC Type VII, and MTA, were found to be equally suitable for VPT, following clinical and radiographic criteria. The success rate with CH (setting) was found to be 93.5%; with GIC (Type VII), it was 97%, and with MTA, it was 100%, respectively.
		GIC VII 79	GIC VII 0.510		
		MTA 84	MTA 0.540		
Holiel et al., 2021 [39]	-	TDMH 98	TDMH 0.235	-	TDMH has a greater potential to induce dentin bridge formation than BD and MTA under standardized conditions, suggesting its suitability as a direct pulp capping material in future clinical applications.
		BD 81	BD 0.147		
		MTA 78	MTA 0.930		
Murugan and han et al., 2021 [40]	Radiopaque structure between the pulp-capping agent and the pulpal space indicates the formation of a dentinal bridge.	-	-	-	MTA exhibited superior performance in dentinal bridge formation when compared to the CH, BD and EERM.
	CH 9 islands of calcified material 6 complete dentinal bridge				
	MTA 6 islands of calcified material 9 complete dentinal bridge				
	BD 8 islands of calcified material 7 complete dentinal bridge				
	ERRM 6 islands of calcified material 9 complete dentinal bridge				
Nowicka et al., 2015 [48]	-	-	CH 0.182 ProRoot MTA 0.230	CH 0.30 *n* = 7 Pro Root MTA 0.45 *n* = 8	In conclusion, the volume of formed reparative dentin bridges depends on the material used for direct pulp capping. BD and MTA induced the formation of bridges with a significantly higher average volume compared with SBU.
			BD 0.212	BD 0.47 *n* = 8	
			SBU 0.030	SBU 0.07 *n* = 2	
**Analysis micro-CT**					
Yoon et al., 2021 [14]	OST + ProRoot MTA showed more mineralized bridge than the ProRoot MTA.	-	-	-	OST can be a supplementary pulp-capping material when used with MTA to make a synergistic effect in hard tissue formation.
Yaemkleebbua et al., 2019 [41]	The MTA and Biodentine exhibited significantly higher BV/TV ratio compared with the CH.	-	-	-	All test materials promoted dentine bridge formation.
Okamoto et al., 2019 [42]	-	-	-	S-PRG and MTA ˜0.05	S-PRG cement is a bioactive material that may be useful in direct pulp capping.
Okamoto et al., 2018 [43]	-	-	-	Pro Root MTA ˜0.045 iRoot BP Plus 0.02	Micro-CT analysis was confirmed as an accurate, objective, and inclusive approach for evaluating quality and quantity of dentin repair.
Kim et al., 2016 [44]	ProRoot MTA homogeneous hard tissue and complete hard tissue bridge. BD calcification tissue exhibiting different degrees of saturation. BA hard tissue, unevenly distributed observed at the pulpal floor and lateral wall of the pulp chamber.	-	-	-	BD and BA could be considered as alternatives to ProRoot MTA.
Ischimoto et al., 2015 [45]	The dentin bridges were uniform in radiopacity.	-	-	-	LiCl has great potential as a bioactive mediating organ-specific trans differentiation.
Hara et al., 2021 [46]	Radiopaque regions covered the exposed pulp surface.	-	-	-	Material that activates canonical Wnt (regulator of the Dentin sialophosphoprotein expression) signalling promotes healing of the pulp–dentin complex.
Al-Hezaimi et al., 2011 [47]	-	-	CH 0.22	-	Tertiary dentin varies in thickness but not in quality, depending on the pulp material used.
			ProRoot 0.43		
Alshwaimi et al., 2016 [49]	-	-	PC 0.4	-	Confirmed the efficacy of MTA for direct pulp capping.
			BG 0.22		
			MTA 0.78		

Abbreviations: BA, BioAggregate cement; BG, betamethasone/gentamicin cream; BD, Biodentine; BV/TV, dentin volume (BV)/tissue volume (TV); CH, calcium hydroxide; DSP, dentin sialoprotein; EERM, EndoSequence root repair material; GIC, glass ionomer cement; iRoot BP Plus, EndoSequence Root Repair Material; LiCl, Lithium chloride; MTA, mineral trioxide aggregate cement; OST, osteostatin; PC, ProRoot white mineral trioxide aggregate, white Portland cement; ProRoot MTA, white mineral trioxide aggregate cement; S-PRG, pre-reacted glass-ionomer; SBU, Single Bond Universal; TDMH, dentin matrix hydrogel; VPT, vital pulp therapy.

**Table 9:** Dentine mineral density (mg/cm^3^) in CBCT and micro-TC analyses in different species.

Source	Nowicka et al., 2015 [48]	Okamoto et al., 2019 [43]	Okamoto et al., 2018 [42]
Imaging technique	CBCT	Micro-CT	Micro-CT
Species	Human	Adult rats	Adult rats
Young Dentin	1600		
Mature Dentin	2300–2500		
Tertiary dentin	1000–1300	1400–1500	1100–1400
